# Ethnobiological study on traditional medicinal plants and fungi recorded in the Naxi Dongba sutras

**DOI:** 10.1186/s13002-021-00459-8

**Published:** 2021-04-29

**Authors:** Haitao Li, Zhiyong Li, Xiaobo Zhang, Shaohua Yang, Cui Chen, Qingning Yang, Chengfeng He, Jianqin Liu, Jingyuan Song

**Affiliations:** 1 Key Lab of Chinese Medicine Resources Conservation, State Administration of Traditional Chinese Medicine of the People’s Republic of China, Institute of Medicinal Plant Development, Chinese Academy of Medical Sciences & Peking Union Medical College, Beijing, 100193 People’s Republic of China; 2 Yunnan Key Laboratory of Southern Medicinal Utilization, Yunnan Branch, Institute of Medicinal Plant Development, Chinese Academy of Medical Sciences & Peking Union Medical College, Jinghong, 666100 People’s Republic of China; 3 School of Pharmacy, Minzu University of China, Beijing, 100081 People’s Republic of China; 4 Yunnan Province Resources of Development and Collaborative Innovation Center for New Traditional Chinese Medicine, Kunming, 650051 People’s Republic of China; 5 State Key Laboratory Breeding Base of Dao-di Herbs, National Resource Center for Chinese Materia Medical, China Academy of Chinese Medical Sciences, Beijing, 100700 People’s Republic of China; 6 Institute of Alpine Economics and Botany, Yunnan Academy of Agricultural Sciences, Lijiang, 674100 People’s Republic of China; 7 Lijiang Medical Association of Minorities, Lijiang, 674100 People’s Republic of China

**Keywords:** Ethnobiology, Lijiang city, The Dongba sutras, Naxi people, Traditional medicine

## Abstract

**Background:**

The Naxi people, living in Southwest China, have a long history and rich characteristic culture. Their ancestors recorded their life practices by ancient hieroglyphs and gradually formed the Dongba Sutras, which, among other knowledge, included the traditional knowledge of Naxi medicine. In the past, most studies on the Dongba Sutras focused on the humanistic culture of Naxi people, whereas studies have rarely focused on Naxi herbal medicinal plants and fungi described in the Dongba Sutras. Studying this aspect is helpful for exploring the traditional culture of Naxi people from the perspective of traditional medicine.

**Methods:**

From February to September 2019, we screened the medicinal plants and fungi from the Dongba Sutras with the help of Dongba. Then, we carried out field investigations and collected voucher specimens of traditional medicinal plants and fungi with the help of 104 Naxi folk healers. The specimens were identified and stored in the Herbarium of Yunnan Branch, Institute of Medicinal Plants, Chinese Academy of Medical Sciences (IMDY). Through semi-structured interviews, we obtained ethnobotanical information of medicinal plants and fungi. The obtained quantitative data were analyzed using the informant consensus factor (ICF) method and the number of citations.

**Results:**

A total of 85 species of medicinal plants and fungi belonging to 51 families and 71 genera were recorded in the Dongba Sutras. Among them, 25 species were endemic to China, and eight species were only distributed in Naxi distribution areas. These medicinal plants and fungi were mainly obtained from the wild, and 22 species could be used as food. The most frequent method of taking medicinal materials was oral-taking after decoction, followed by topical and sometimes buccal. The methods of processing these medicinal materials included water decoction, warm water flushing, and drinking after soaking. The medicinal plants and fungi in the Dongba Sutras are used to treat 96 conditions classified into 13 disease groups according to the International Classification of Primary Care second edition. Further analysis indicated that most of these species were utilized for treating diseases from the digestive (D) group, followed by those from the respiratory (R) group, musculoskeletal (L) group, general, and unspecified (A) group. Moreover, the Naxi people have a high consensus on the treatments of diseases from these four pathological groups.

**Conclusions:**

The Naxi traditional medicine is characterized by simple materials, easy operation, and distinctive national characteristics. The ancient Naxi people recorded their highly developed medical culture in the Dongba Sutras. Natural plant resources found around them were their primary choices for both medicine and diet therapy. The ecological ethics of Naxi people have positive significance for the conservation of wild resources in their area.

## Background

The Naxi people inhabit areas of Southwest China, and they have a long history and a rich characteristic culture. Dongba symbols are the only hieroglyphs in the world that are still in use [1]. Joseph F. Rock collected about 8000 copies of Dongba scriptures, which were later deposited in major European and American libraries. Since his book was titled *The Ancient Na-Khi Kingdom of Southwest China* [2], Naxi people and their Dongba culture are famous throughout the world. The Dongba Sutras have become the main written materials for studying the Dongba culture. The Dongba Sutras is a special scripture and different from Buddhism Sutras or other classics. The content of Dongba Sutras covers the history, philosophy, society, religion, language and script, music, art, dance, and many other traditional subjects related to the Dongba culture. It is praised by academic circles as “the encyclopedia of ancient Naxi people” [3]. Naxi medical culture is an important part of Dongba culture. The Dongba Sutras contain information about the unique medical culture of the Naxi people, and they are the most important documents for studying Naxi medicine. The name “Dongba” is the appellation of the Naxi religious clergy and can be translated as “the wise.” They are senior intellectuals and the main inheritors of the Dongba culture of the Naxi people, and most of them are skilled in singing, dancing, calligraphy, history, painting, and medicine.

Naxi ancestors have rich medical experience in the practice of fighting against diseases, and they created “Naxi medicine” or “Dongba medicine” [4]. These traditional medical experiences have been recorded by the Naxi people in the form of hieroglyphs, and they formed the Dongba Sutras. Only the Dongba who as the clergyman can recognize the hieroglyphics of Dongba sutras, and they lack of scientific research methods including ethnobotany. Therefore, in the existing literature, the medicinal plants and fungi recorded in Dongba sutras rarely corresponded to their scientific names.

Due to historical reasons, a large number of Dongba scriptures have been lost, some of them are scattered abroad or collected by privates. Currently, there are about 30,000 volumes of the Dongba Sutras, which are mainly stored in museums and libraries in China, the USA, Germany, France, Great Britain, and other countries [3]. These sutras are based on extensive experience in treating diseases and provide great knowledge of medicine. *Chien Song Lü* and *Chongren Pandi to Find Medicine* are the most representative sutras [5]. *Chien Song Lü* is the only medical book written in hieroglyphs of the Naxi people, and it includes data on dozens of medicinal plants. *Chongren Pandi to Find Medicine* includes records of the traditional treatment methods, the morphology, and function of some medicinal plants, and it has important reference value for the current medical practice [5]. The publication entitled *The Complete Works of Dongba Sutras in Naxi* [6] lays the foundation for deciphering the mysterious Naxi Dongba medicine.

In addition to the Dongba Sutras, in Naxi culture, a lot of valuable traditional knowledge has been transmitted orally, including a lot of precious medical information. Therefore, Naxi culture still needs to be further studied and systematically organized [4]. In recent decades, ethnomedicinal knowledge in Naxi communities has lost rapidly along with the high-speeded development of the Chinese economy. In particular, Lijiang is a famous tourism destination, and few young generations study traditional medicinal knowledge from the old generation. Less and less Naxi people use (or even recognize) traditional medicinal plants. Thus, it becomes very urgent and necessary to study medicinal plants recorded in the Dongba Sutras.

## Materials and methods

### Study area

Lijiang is a prefecture-level city in Yunnan Province, Southwest China. It is located in Hengduan Mountains, between 25° 23′–27° 56′ N and 99° 23′–101° 11′ E. The total area of Lijiang City covers 20 600 km^2^ [7]. The terrain of the area is high in the northwestern part and low in the southeastern part, with the highest altitude of 5596 m and the lowest altitude of 1015 m. The maximum altitude difference of Lijiang is 4581 m [8].

The climate of Lijiang belongs to subtropical humid climate [9]. There is abundant rainfall and a distinct dry and wet season. The average annual rainfall is about 1 000 mm, and the rainy season lasts from May to October being particularly pronounced in July and August. The annual average temperature is between 13 °C and 20 °C, the average temperature of the hottest month is 18–26 °C, and the average temperature of the coldest month is 4–12 °C. Lijiang has 2500 h of annual sunshine and 147 kcal/cm^2^ of annual solar radiation [9].

Lijiang has a forest coverage rate of 70%. The area is rich in medicinal materials and other exploitable biological resources and is known as the “kingdom of alpine plants” and “hometown of medicinal materials” [8].

The key areas of the present study were Gucheng District and Yulong County in Lijiang city, Yunnan Province, China. This area is the most concentrated area of the Naxi population in the world, with about 210 000 people, accounting for 68.5% of the total Naxi population. Naxi people live in mountainous areas with inconvenient transportation and abundant biological resources, which is why their tradition is the most convenient mean of resisting diseases. At the same time, the inheritance model of Dongba culture is masters teaching apprentices that makes a better inheritance of the Naxi traditional medicinal culture.

### Data collection

From February to September 2019, we screened the medicinal plants and fungi from the Dongba Sutras with the help of Dongba (the clergies who can read and write hieroglyphs) and translate the hieroglyphs into the Naxi language. Then, we carried out field research with assistance from 104 Naxi folk healers and collected traditional medicinal plant specimens. The basic survey information such as age and gender was collected and recorded. Using semi-structured interviews [10], ethnobotanical knowledge was obtained, including information about the local name, medicinal parts, harvesting methods, preparation methods, and indications of the medicinal plants and fungi from the Dongba Sutras. The informed consent of the participants was obtained before conducting the interviews, and the ethical guidelines prescribed by the International Society of Ethnobiology [11] were followed. The local names were transliterated from the Naxi or local Chinese pronunciation into the Roman alphabet following the Scheme for the Chinese Phonetic Alphabet [12] and the Basic Rules for Hanyu Pinyin Orthography [13]. The diseases treated by the medicinal plants and fungi from the Dongba Sutras were classified according to the International Classification of Primary Care (ICPC-2) [14] of the WHO (World Health Organization) [15, 16].

### Plant materials

With the help of Naxi folk healers, 3–5 specimens of each species were collected, and the information about their habitats (e.g., altitude, latitude, longitude, and vegetation type), plant morphology (e.g., plant height, color of flowers, and corolla type), and date of the collection were recorded. The scientific and Chinese names were recorded on the label. These specimens were stored at the Herbarium, Yunnan Branch, Institute of Medicinal Plants, Chinese Academy of Medical Science (IMDY).

### Plant identification

The following literature was used to identify the family and species names of the collected plants: *Flora of China* [17], *Flora Reipublicae Popularis Sinicae* [18], and *Flora Yunnanica* [19]. The scientific names were checked on The Plant List website [20]. All the plants listed are sorted at family level circumscription follows APG IV [21].

### Data analysis

The data obtained in this study were analyzed using Microsoft Office Excel (2010) spreadsheet software. Quantitative data analysis was conducted using the informant consensus factor (ICF) method and the number of citations. ICF was calculated as ICF = (Nur - Nt)/(Nur - 1), where Nur is the sum of plant species used by all the respondents to treat a particular disease, and Nt is the number of identical plant species used by all the respondents to treat a particular disease [22].

## Results and discussion

### Demographic features of the respondents

A total of 104 respondents were interviewed (Table 1). Among them, male respondents highly outnumbered the female respondents, and 79.81% of them were over 50 years old. Naxi people live in mountainous areas and commonly collect medicinal plants. In this harsh environment, men have an advantage over women due to their physical abilities. Because the experience of treating diseases is based on long-term practice, the medical experience mastered by older healers is more comprehensive and reliable than those learned by younger healers. Moreover, it ensures the reliability of the knowledge obtained in this survey.

**Table 1:** Demographic features of the respondents

Demographic features	Number	Proportion (%)
**Age**		
31–40	7	6.73
41–50	14	13.46
51–60	28	26.92
61–70	24	23.08
71–80	24	23.08
81 and above	7	6.73
**Sex**		
Female	4	3.85
Male	100	96.15
**Education level**		
Illiterate	10	9.62
Primary school	61	58.65
Junior middle school	12	11.54
Senior middle school	8	7.69
Teacher training school	1	0.96
School of health	2	1.92
Polytechnic school	5	4.81
Junior college	4	3.85
University	1	0.96
**Nationality**		
Naxi	74	71.15
Lisu	16	15.38
Han	5	4.81
Zang	5	4.81
Bai	3	2.88
Yi	1	0.96
**Ways of learning medicine**		
Ancestral	73	70.19
Ancestral,^*^master	8	7.69
Master	7	6.73
Ancestral, self-taught	6	5.77
Master, self-taught	4	3.85
Ancestral, learning at school	2	1.92
Ancestral,self-taught, learning at school	1	0.96
Master, learning at school	1	0.96
Master,self-taught, learning at school	1	0.96
Self-taught	1	0.96

*Master: an authority qualified to teach apprentices

The educational level of the respondents was generally low, and most of them had no higher education. However, this did not affect the reliability of the results, because the acquired traditional knowledge has truly maintained the characteristics of the Naxi people.

The respondents were mainly Naxi (71.158%), followed by the Lisu (15.38%). Other ethnic groups included Han, Tibetan, Bai, and Yi. All of these people lived in Naxi communities, and their medical skills were learned from Naxi healers. All the respondents were folk healers. Although there are many ways to learn medical skills, most respondents (70.19%) developed their medical experiences with the help of their ancestors. None of the respondents had regular jobs, and many of them were local Dongba who were priests and folk healers.

### Diversity of medicinal plants and fungi in the Dongba sutras

According to our investigation, a total of 85 species of medicinal plants and fungi belonging to 51 families and 71 genera were recorded in the Dongba Sutras (Table 2). In the middle and high altitude areas, the main tree species belonged to the families Pinaceae, Cupressaceae, Ericaceae, and Fagaceae. Almost all parts of these plants can be used as medicine, especially their branches, which are often used by Naxi priests for various sacrificial activities. The highest numbers of plant species recorded belonged to the families Asteraceae (six species) and Polygonaceae (six species), followed by the Rosaceae (four species). It is worth mentioning that from the genus *Rheum* alone, we recorded three species. In addition to *Rheum officinale* recorded in the Pharmacopoeia of People’s Republic of China [23], we also recorded *R. delavayi* and *R. likiangense*, but their usage was different from that of *R. officinale* recorded in the Pharmacopoeia of People’s Republic of China.

**Table 2:** Number of medicinal plants and fungi contained in the Dongba Sutras

Category	Number of families	Number of genera	Number of species
Fungi	3	3	3
Pteridophyta	3	3	3
Gymnospermae	2	3	5
Angiospermae	43	62	74
Total	51	71	85

Of all recorded species, herbaceous plants (49 species) accounted for the greatest number (Table 3), followed by trees (21 species) and shrubs (5 species). As herbaceous plants can more easily survive in a new environment than trees and shrubs [24], especially in the alpine mountains inhabited by the Naxi people, there is a lack of diversity of tree species, whereas the low herbaceous plants were abundant. At the same time, herbaceous plants are more convenient to collect than other plant life forms. Thus, the utilization rate of herbaceous plants is higher than that of trees and shrubs.

**Table 3:** Habits of medicinal plants and fungi contained in the Dongba Sutras

Living habits	Number of species	Proportion (%)
Herbs	46	54.12
Trees	21	24.71
Shrubs	5	5.88
Woody vines	4	4.71
Climbing shrubs	3	3.53
Herbaceous climbers	3	3.53
Macro-fungi	3	3.53
**Total**	**85**	**100.00**

The medicinal parts of 85 medicinal plant and fungus species used by the respondents are indicated in Tables 4 and 10. The Naxi people knew that different medicinal parts have different effects. According to our analysis, in addition to the plant’s medicinal efficacy, the difficulty of its collection also affects which parts would be used. The Naxi people preferred to collect easily collectible plant parts as raw materials for medicinal preparations. Among plant life forms, herbs and small shrubs are most commonly used as medicines, and the respondents reported that for this purpose, they used whole plants, roots, or rhizomes, whereas when trees, big shrubs, or woody vines are used for medicinal preparations, the respondents used stems, branches, leaves, or bark. The flowering and fruiting periods of these plants are short; therefore, their fruits, seeds, flowers, and buds are seldom used as medicinal parts. Plant secretions are rarely used as medicinal materials because of the difficulty of their collection.

**Table 4:** Medicinal parts of plants and fungi recorded in the Dongba Sutras

Medicinal parts	Number of species	Proportion (%)
Roots or rhizomes	29	24.37
Whole plants	22	18.49
Leaves	19	15.97
Stems or branches	17	14.29
Fruits or seeds	9	7.56
Flowers or flower buds	8	6.72
Bark	8	6.72
Aerial parts	3	2.52
Fungi (fruit body)	3	2.52
Secretions	1	0.84
Total	119	100.00

Note: One or more parts of the same plant can be used as medicine, which is why the total number of medicinal parts exceeds the total number of species

Most of the medicinal plants in the Dongba Sutras are common plants in the studied area. The abundance of medicinal plants, determined according to the classification of abundance by Germany Ecologist Oscar Drude [25], is shown in Table 5. According to this classification, the highest number of species used by the respondents is forest species, such as *Quercus aquifolioides*, *Q. aliena* var. *acuteserrata*, *Populus rotundifolia* var. *bonatii*, and *Pinus yunnanensis*. The group with few or dispersed organism included only three species: *Poria cocos*, *Dobinea delavayi*, and *Panax japonicus* var. m*ajor*. Although the medicinal materials from these species are rarely found in the wild, they have been cultivated in the area and thus have been successfully used as medicines.

**Table 5:** Abundance of medicinal plants and fungi contained in the Dongba Sutras

Abundance*	Number of species	Proportion (%)
Soe	4	4.71
Cop3	13	15.29
Cop2	17	20.00
Cop1	38	44.71
Sp	10	11.76
Sol	3	3.53
Un	0	0.00
Total	85	100.00

*Soe (Sociales): High number of individuals, the above-ground plant part is closed

Cop3 (Copiosae): High number of individuals, but the above-ground plant part is not closed

Cop2: Large and common plants

Cop1: Large plants, but small populations

Sp (Sparsal): Low number of plants, scattered

Sol (Solitariae): Low number of plants, sparse

Un (Unicum): Only one individual

Since ancient times, Naxi people have lived in mountainous areas, where transportation is inconvenient. The medicines they used were collected in the mountains, and rare medicinal plants were cultivated in their courtyards in order to be convenient for collection. Therefore, the medicinal plants described in the Dongba Sutras were mainly wild plants, accounting for 76.47% of all medicinal plants described in the Dongba Sutras (Table 6). Because of the small population of Naxi people, their use of wild medicinal plants does not present a threat to the stability of wild plant populations.

**Table 6:** Sources of drugs contained in the Dongba Sutras

Sources	Number of species	Proportion (%)
Wild	65	76.47
Cultivated	7	8.24
Mixture of wild and cultivated	13	15.29
Total	85	100.00

Food therapy is an important characteristic of Chinese culture and traditional Chinese medicine (TCM). “One Root of medicine and food” is a summary of the Chinese people’s understanding of medicine and food and their relationship [26]. The life of the Naxi people is closely related with medical dietary plants, and their medicinal diets are indispensable to the health of their communities [27]. Among the medicinal plants in the Dongba Sutras, 22 species can be consumed as vegetables, fruits, dried fruits, or condiments (Table 7). For example, *Lagenaria siceraria*, *Brassica rapa*, *Foeniculum vulgare*, and *Allium ascalonicum* are common vegetable species. *Setaria italica* var. *germanica* is also used as food. For a long time, the Naxi people considered that these foods and vegetables can be used to treat and prevent diseases. Thus, they recorded them in the Dongba Sutras. Some of these medicines are used to prepare tea and do not have any negative side effects. For example, the aerial parts of *Elsholtzia rugulosa* which has the effect of relieving summer heat. The plant as a substitute for tea is easy to collect and prepare and has widely been used by the Naxi people. This indicated that in the Naxi people, maintaining a healthy daily diet is a very important factor in disease prevention.

**Table 7:** List of medicinal and edible plants and fungi recorded in the Dongba Sutras

ID	Family	Scientific name	Resource type	Food type
1	Amaranthaceae	*Amaranthus hypochondriacus*	Wild	Vegetable
2	Amaryllidaceae	*Allium ascalonicum*	Cultivated	Vegetable
3	Amaryllidaceae	*Allium hookeri*	Cultivated, wild	Vegetable
4	Amaryllidaceae	*Allium sativum*	Cultivated	Vegetable
5	Apiaceae	*Foeniculum vulgare*	Cultivated	Vegetable
6	Brassicaceae	*Brassica rapa*	Cultivated	Vegetable
7	Cactaceae	*Opuntia ficus-indica*	Cultivated, wild	Fruit
8	Cannabaceae	*Cannabis sativa*	Cultivated, wild	Condiment
9	Cucurbitaceae	*Lagenaria siceraria*	Cultivated	Vegetable
10	Ebenaceae	*Diospyros lotus*	Cultivated, wild	Fruit
11	Fabaceae	*Pueraria montana* var. *chinensis*	Wild	Beverage
12	Juglandaceae	*Juglans regia*	Cultivated, wild	Dry fruit
13	Lamiaceae	*Elsholtzia rugulosa*	Wild	Beverage
14	Lauraceae	*Neocinnamomum delavayi*	Wild	Condiment
15	Pinaceae	*Pinus armandii*	Wild	Dry fruit
16	Poaceae	*Setaria italica* var. *germanica*	Cultivated	Food
17	Polyporaceae	*Poria cocos*	Wild	Vegetable
18	Rosaceae	*Prunus mume*	Cultivated, wild	Fruit
19	Rosaceae	*Rubus biflorus*	Wild	Fruit
20	Rosaceae	*Rubus coreanus* var. *tomentosus*	Wild	Fruit
21	Rosaceae	*Rubus niveus*	Wild	Fruit
22	Schizophyllaceae	*Schizophyllum commune*	Wild	Vegetable

### Medicine preparation methods and applications

**Fig. 1:**
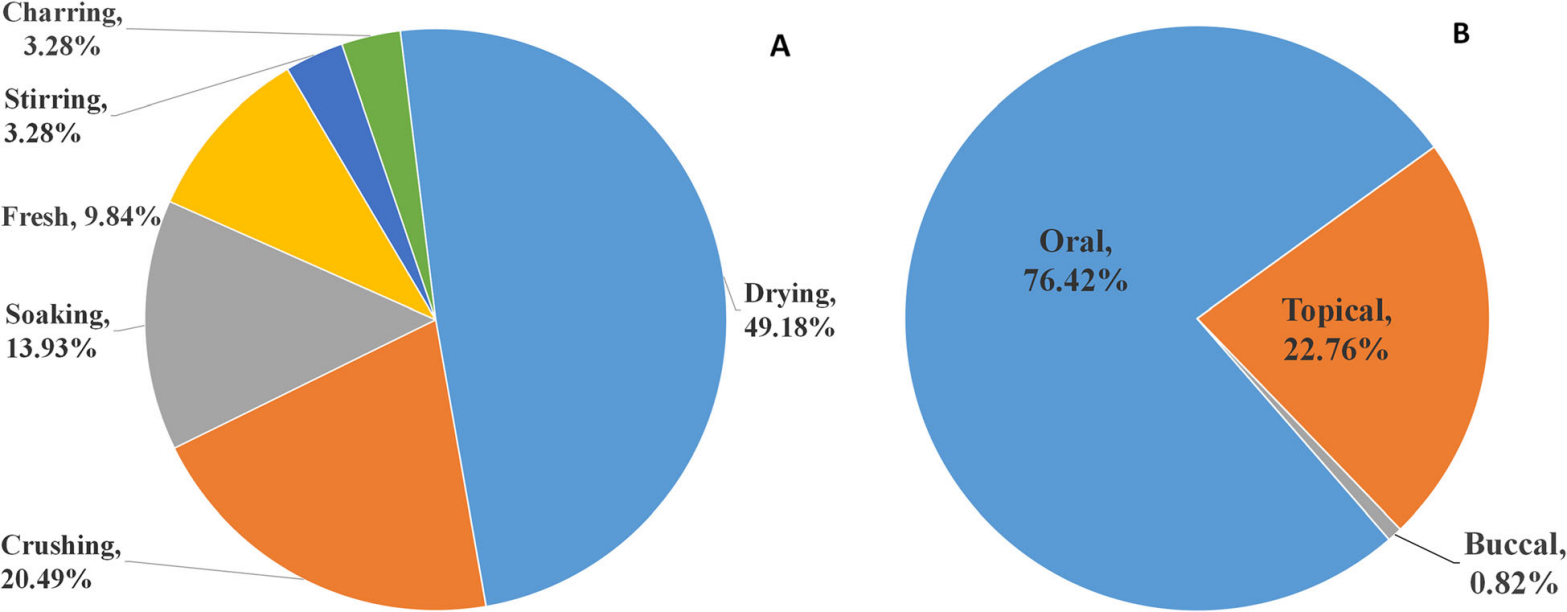
**a** Preparation methods of medicinal materials. **b** Medicine administration methods used by the Naxi people

The folk preparation methods of traditional Naxi medicine were relatively simple (Fig. 1a); most of them included washing and direct drying of the plant material (49.18%), followed by crushing (20.49%), soaking (13.93%), using fresh products (9.84%), blending with other agents (3.28%), and carbonization (3.28%). The medium used in the soaking process was mainly wine or water, whereas the medium used in blending included edible oils, vinegar, and honey, etc. The use of fresh plant parts as medicine is characteristic of Naxi medicine because this method is simpler to use than other methods. In this method, the medicinal parts are removed from the plants and washed, and they are used after mashing or chewing. In addition, juice extracted directly from the plant is also a common method of fresh plant intake and is mostly used for topical application. The main method of medicine consummation was oral, followed by topical and rarely buccal (Fig. 1b). Oral administration included three methods: boiling in water, washing in warm water, and drinking after soaking.

### ICF, conditions, and diseases treated by the studied plants and fungi

The informant consensus factor (ICF) is a measure of information diversity. The higher the ICF value, the greater the difference among plant species used in the treatment of a given disease, and the lower the ICF value, the smaller the difference among plant species used in the treatment of a disease [22]. We found that the medicinal plants and fungi in the Dongba Sutras are used to treat 96 conditions, which can be classified into 13 disease groups according to ICPC-2 (Table 8 and Table 9). The highest ICF values were recorded for the eye group (F), cardiovascular group (K), and psychological group (P) (ICF = 1.50), followed by the neurological group (N), female genital group (X), and male genital group (Y) (ICF: 1.00). Among the medicinal plants provided by different respondents, there are very few (only one or none) identical plants that can be used to treat the same group of diseases. This showed that there are many differences among Naxi people in the methods of treating a specific disease, i.e., that they have low consensus about disease treatment methods. There are two possible reasons for this: (1) as the Naxi people live in biodiversity-rich areas, the abundant medicinal plant resources provided them with a wide choice of medicinal plants to use [28], and (2) different Naxi folk healers may have different degrees of understanding of the same disease (e.g., some may be focused more on the symptoms of a disease, but ignore or miss the real cause of the disease).

**Table 8:** Informant consensus factor (ICF) values of the medicinal plants and fungi contained in the Dongba Sutras

Disease types	The sum of plant species (Nur)	The number of identical plant species used (Nt)	ICF
A: General and unspecified	21	6	0.75
D: Digestive	36	15	0.60
F: Eye	3	0	1.50
K: Cardiovascular	3	0	1.50
L: Musculoskeletal	21	12	0.45
N: Neurological	4	1	1.00
P: Psychological	3	0	1.50
R: Respiratory	29	13	0.57
S: Skin	11	2	0.90
T: Endocrine/metabolic and nutritional	1	1	-
U: Urological	12	4	0.73
X: Female genital	16	1	1.00
Y: Male genital	14	1	1.00

**Table 9:** Ethnomedicinal data of medicinal plants and fungi recorded in the Dongba Sutras

Chinese name	Naxi name	Scientific name	Family/voucher specimen/habitat^**a**^/habit^**b**^	Part used	Preparation method	Route of administration	Diseases treated/number of respondents (ICPC-2)
Zhu Sheng Rou Qiu Jun	Men Mu	*Engleromyces goetzi* P. Henn.	Hypocreaceae/NX0759/W/M	Fruit body	Drying	Oral	Elevated Blood Pressure K85 (57)Headache N01 (64) Throat Symptom R21 (52)
Lie Zhe Jun	Si Du Mou Pei	*Schizophyllum commune* Franch.	Schizophyllaceae/NX0360/W/M	Fruit body	Drying	Oral	Cough R05 (104) Pleurisy/Pleural Effusion R82 (104)
Fu Ling	Tuo Ken Liu	*Poria cocos* (Schw.) Wolf	Polyporaceae/NX0581/W/M	Fruit body	Drying/Soaking	Oral/Topical	Gonorrhoea Female X71 (12) Limited Function/Disability (L) L28 (68)
Dian Zhuang Juan Bai	Ci Liu Liu Ru Da Bie	*Selaginella pulvinata* (Hook. et Grev.) Maxim.	Selaginellaceae/NX0281/W/H	Roots	Charring	Oral	Bleeding/Hemorrhage NOS A10 (76)
Jie Jie Cao	Mie Liu Ku Sa	*Equisetum ramosissimum* Desf.	Equisetaceae/NX0364, NX0657/W/H	Whole plants	Charring/Drying	Oral	Eye Discharge F03 (80) Genital Disease Male other Y99 (46) Menstruation Excessive X06 (72)
Chuan Dian Hu Jue	Lu Ba Di Li	*Drynaria delavayi* Christ	Drynariaceae/NX0151/W/H	Rhizomes	Crushing	Topical	Fracture: Femur L75 (78) Fracture: Hand / Foot Bone L74 (64) Fracture: Other L76 (43) Fracture: Radius/Ulna L72 (37) Fracture: Tibia/Fibula L73 (90) Limited Function/Disability (L) L28 (89) Musculoskeletal Disease other L99 (44) Osteoarthrosis other L91 (37) Pelvis Symptom/Complaint Female X17 (18)
Li Jiang Yun Shan	Li Ben Le	*Picea likiangensis* (Franch.) Pritz	Pinaceae/NX0318/W/T	Fruits	Drying	Oral	Osteoarthrosis other L91 (102) Rheumatoid/Seropositive Arthritis L88 (99)
Hua Shan Song	Se Tong	*Pinus armandii* Franch.	Pinaceae/NX0223, NX0322/W/T	Secretion	Drying	Oral	Constipation D12 (25) Cough R05 (52) Epilepsy N88 (37)
Yun Nan Song	Ge Bo Ha	*Pinus yunnanensis* Franch.	Pinaceae/NX0159/W/T	Flowers, branches	Crushing/stirring	Oral	Acute Bronchitis/Bronchiolitis R78 (75) Chronic Bronchitis R79 (84) Limited Function/Disability (L) L28 (28) Pneumonia R81 (76) Tuberculosis A70 (23)
Gan Xiang Bai	Xiong Ban	*Cupressus duclouxiana* Hickel	Cupressaceae/NX0558/W/T	Branches, leaves	Drying	Topical	Leg/Thigh Symptom L14 (87) Low Back Symptom L03 (68) Muscle Pain L18 (104)
Gao Shan Bai	Xiu Xu	*Juniperus squamata* Buch.-Ham. ex D.Don	Cupressaceae/NX0257, NX0614/W/S or T	Branches, leaves	Drying	Oral/Topical	Intermenstrual Bleeding X08 (100) Menstruation Irregular/Frequent X07 (86)
Hong Hua Wu Wei Zi	Gua Ji Liu	*Schisandra rubriflora* Rehder et E.H.Wilson	Schisandraceae/NX0248/W/WV	Bark	Soaking	Oral	Abdominal Pain Localized other D06 (44) Cystitis/Urinary Infection other U71 (32) Pain General/Multiple Sites A01 (104) Sleep Disturbance P06 (46) Trauma/Injury A80 (104)
Shan Yu Lan	Han Yi Ba Da	*Magnolia delavayi* Franch.	Magnoliaceae/NX0701/C/T	Flowers	Soaking	Oral	Abdominal Pain Epigastric D02 (53)
Xin Zhang	Sei Bi	*Neocinnamomum delavayi* (Lec.)H. Liu	Lauraceae/NX0760/W/T	Leaves, bark	Stirring	Topical	Diarrhoea D11 (45)
Chang Pu	Ji Chu Buer	*Acorus calamus* Linn.	Acoraceae/NX0116/C, W/H	Whole plants	Crushing	Oral	Abdominal Pain Epigastric D02 (99) Influenza R80 (104) Mumps D71 (104) Upper Respiratory Infection Acute R74 (58)
Dong Fang Ze Xie	He Ke Gu	*Alisma orientale* (Samuel.) Juz.	Alismataceae/NX0520/C,W/H	Roots	Drying	Oral	Dysuria/Painful Urination U01 (72)
Shou Shen	A You La Ba	*Gymnadenia conopsea* (Linn.) R. Br.	Orchidaceae/NX0352/W/H	Roots	Crushing/drying	Oral	Cough R05 (74) Low Back Symptom L03 (45) Pain General/Multiple Sites A01 (55) Sexual Function Symptom/Complaint Male Y08 (31)
Xi Nan Shou Shen	A You La Ba	*Gymnadenia orchidis* Lindl.	Orchidaceae/NX0349/W/H	Roots	Crushing/drying	Oral	Cough R05 (74) Low Back Symptom L03 (45) Pain General/Multiple Sites A01 (55) Sexual Function Symptom / Complaint Male Y08 (31)
Shou Cao	Lu Bu Ge	*Spiranthes sinensis* (Pers.) Ames	Orchidaceae/NX0122, NX0544/W/H	Whole plants	Crushing/soaking/stirring	Oral/Topical	Herpes Zoster S70 (53) Low Back Symptom L03 (86) Pain General/Multiple Sites A01 (97) Sexual Function Symptom/Complaint Male Y08 (24)
Huo Cong	Cong Ke Pei Er	*Allium ascalonicum* Linn.	Amaryllidaceae/NX0746/C/H	Whole plants	Drying	Oral	Influenza R80 (69) Upper Respiratory Infection Acute R74 (104)
Kuan Ye Jiu	Ju Ge Shu	*Allium hookeri* Thwaites	Amaryllidaceae/NX0705/C, W/H	Leaves, roots	Fresh	Topical	Allergy/Allergic Reation A92 (93)
Suan	Gu	*Allium sativum* Linn.	Amaryllidaceae/NX0764/C/H	Whole plants	Drying/fresh	Oral/Topical	Animal/Human Bite S13 (98) Insect Bite/Sting S12 (104)
Mi Chi Tian Men Dong	Ai Xu	*Asparagus meioclados* Lévl.	Asparagaceae/NX0640/W/H	Roots	Drying	Oral	Cough R05 (104) Respiratory Disease other R99 (69)
Ji Xiang Cao	Gu Ke Gu Zhe Le	*Reineckea carnea* (Andr.) Kunth	Asparagaceae/NX0651/W/H	Whole plants	Crushing/fresh/drying	Oral/Topical	Acute Bronchitis/Bronchiolitis R78 (101) Chronic Bronchitis R79 (103) Cystitis/Urinary Infection other U71 (45) Fracture: Femur L75 (79) Fracture: Hand/Foot Bone L74 (65) Fracture: Other L76 (47) Fracture: Radius/Ulna L72 (53) Fracture: Tibia/Fibula L73 (100) Genital symptom/Complaint Female other X29 (36) Low Back Symptom L03 (100) Pain General/Multiple Sites A01 (66)
Dian Jiang Hua	Gu Shu	*Hedychium yunnanense* Gagnep.	Zingiberaceae/NX0610/W/H	Roots	Drying/fresh	Oral/Topical	Influenza R80 (88) Orchitis/Epididymitis Y74 (18) Osteoarthrosis other L91 (64) Rheumatoid/Seropositive Arthritis L88 (59) Upper Respiratory Infection Acute R74 (74)
Chang Yuan Qiao Jian Zhu	Ju Me	*Fargesia orbiculata* T. P. Yi	Poaceae/NX0665/W/S	Leaves	Charring	Oral	Influenza R80 (58) Trauma/Injury A80 (28) Upper Respiratory Infection Acute R74 (79)
Su	Chong Jing	*Setaria italica* var. *germanica* (Mill.) Schred.	Poaceae/NX0765/C/H	Whole plants	Drying	Oral	Dyspepsia/Indigestion D07 (35)
Jin Mao Tie Xian Lian	Hai Ke Si Zi Beng	*Clematis chrysocoma* Franch.	Ranunculaceae/NX0370/W/WV	Whole plants	Drying	Oral	Bladder Symptom U13 (67)
He Bing Tie Xian Lian	Ze Die Ba	*Clematis connata* DC.	Ranunculaceae/NX0721/W/WV	Stem	Drying	Oral	Pelvis Symptom/Complaint Female other X17 (39)
Pao Hua Shu	Gai Si Ze	*Meliosma cuneifolia* Franch.	Sabiaceae/NX0669/W/T	Leaves, stem	Drying	Oral	Cystitis/Urinary Infection other U71 (15)
Chuan Dian Que Er Dou	Wen Lu Ban Qi Shi	*Chesneya polystichoides* (Hand.-Mazz.) Ali	Fabaceae/NX0265, NX0691/W/H	Roots	Soaking	Oral	Weakness/Tiredness General A04 (85)
Fen Ge	Gai Gan Er	*Pueraria montana* var. *chinensis* (Ohwi) Sanjappa et Pradeep	Fabaceae/NX0632/W/SC	Roots, flowers	Drying	Oral	Elevated Blood Pressure K85 (86) Headache N01 (103) Neck Symptom L01 (74) Pneumonia R81 (41) Vertigo/Dizziness N17 (104)
Mei	Se Ka Hao	*Prunus mume* Siebold et Zucc.	Rosaceae/NX0435/C, W/T	Fruits	Charring/drying	Topical/Oral	Abdominal Pain D01 (104) Asthma R96 (77) Diarrhea D11 (86) Nose Bleed/Epistaxis R06 (104)
Fen Zhi Mei	Qi Pa Ke	*Rubus biflorus* Buch.-Ham. ex Sm.	Rosaceae/NX0145, NX0552/W/CS	Roots, branches, leaves	Drying	Oral	Menstruation Irregular/Frequent X07 (67)
Mao Ye Cha Tian Pao	Qi Dong Bei	*Rubus coreanus* var. *tomentosus* Card.	Rosaceae/NX0661/W/CS	Roots	Drying	Oral	Cystitis/Urinary Infection other U71 (100) Menstruation Irregular/Frequent X07 (67) Prostate Symptom Y06 (53) Urinary Calculus U95 (99)
Hong Pao Ci Teng	A He Le De Ken	*Rubus niveus* Thunb.	Rosaceae/NX0461, NX0659/W/CS	Roots, leaves, fruits	Drying	Oral	Cystitis/Urinary Infection other U71 (94)Menstruation Irregular/Frequent X07 (67) Prostate Symptom Y06 (53) Urinary Calculus U95 (99)
Zhou Zhi Shu Li	Qi Na Ze	*Rhamnus virgata* Roxb.	Rhamnaceae/NX0655/W/T	Leaves, branches	Drying	Oral	Malignancy A79 (86)
Da Ma	Sa	*Cannabis sativa* Linn.	Cannabaceae/NX0561, NX0630/C,W/H	Fruits, leaves, stem, bark	Crushing/drying	Oral	Constipation D12 (46)
Rui Chi Hu Li	La Ze	*Quercus aliena* var. *acutidentata* Maxim. ex Wenz.	Fagaceae/NX0646/W/T	Branches, leaves	Drying	Oral	Osteoarthrosis other L91 (101) Rheumatoid/Seropositive Arthritis L88 (104)
Chuan Dian Gao Shan Li	Bei Shi	*Quercus aquifolioides* Rehd. et Wils.	Fagaceae/NX0241/W/T	Fruits, bark, flowers	Drying	Oral	Nose Bleed/Epistaxis R06 (55) Viral Hepatitis D72 (66)
Hu Tao	Gu Du Bai Duo	*Juglans regia* Linn.	Juglandaceae/NX0570/C, W/T	Bark	Soaking/drying	Oral	Cholecystitis/Cholelithiasis D98 (63) Diabetes Insulin Dependent T89 (75) Diabetes Non-Insulin Dependent T90 (69) Dyspepsia/Indigestion D07 (90) Influenza R80 (79) Upper Respiratory Infection Acute R74 (81)
Hu Lu	Bei Pu Gu De	*Lagenaria siceraria* (Molina) Standl.	Cucurbitaceae/NX0675/C/HV	Leaves	Drying	Oral	Genital Disease Male other Y99 (68)
Mao Gua	Bu Luo Lan	*Solena amplexicaulis* (Lam.) Gandhi	Cucurbitaceae/NX0763/W/HV	Roots	Fresh	Topical	Burn/Scald S14 (22) Cough R05 (45)
Wu Bing Jin Si Tao	Ni Mei Hei Tu Ba	*Hypericum augustini* N. Robson	Hypericaceae/NX0142/W/WV	Whole plants	Crushing/drying	Oral/Topical	Acute Hepatitis A D73 (75) Dyspepsia/Indigestion D07 (48) Genital Disease Male other Y99 (90) Gonorrhoea Male Y71 (101) Pain General/Multiple Sites A01 (12) Prostate Symptom Y06 (96) Psoriasis S91 (42) Pyelonephritis/Pyelitis U70 (21) Viral Hepatitis D72 (59)Worms/Other Parasites D96 (97)
Dian Nan Shan Yang	La Ka	*Populus rotundifolia* var. *bonatii* (H. Lévl.) C. Wang & S. L. Tung	Salicaceae/NX0672/W/T	Bark	Soaking/drying	Oral/Topical	Infectious Disease A78 (88) Viral Disease A77 (79) Worms/Other Parasites D96 (22)
Chui Liu	Re Pei	*Salix babylonica* Linn.	Salicaceae/NX0555/W/T	Branches, leaves, roots	Fresh	Oral	Teeth/Gum Symptom D19 (17)
Qiu Hua Liu	Ji Re	*Salix variegata* Franch.	Salicaceae/NX0563/W/T	Branches, leaves	Drying	Oral	Haematuria U06 (97) Urinary Calculus U95 (79) Viral Hepatitis D72 (45)
Zi Di Yu	Qie Sai Che E	*Geranium strictipes* R. Knuth	Geraniaceae/NX0378/W/H	Roots	Crushing/drying	Oral	Dyspepsia/Indigestion D07 (82) Mumps D71 (62) Pneumonia R81 (75) Viral Hepatitis D72 (48)
Yang Jiao Tian Ma	Ju Luo Lan	*Dobinea delavayi* (Baill.) Baill.	Anacardiaceae/NX0762/W/H	Roots	Crushing	Oral	Limited Function/Disability (L) L28 (53)
Chuan Dian Wu Huan Zi	Ba De Zi	*Sapindus delavayi* (Franch.) Radlk.	Sapindaceae/NX0125/C, W/T	Fruits	Drying	Oral	Dyspepsia/Indigestion D07 (25)
Chuan Lian	Da Liu Liu	*Melia toosendan* Sieb. et Zucc.	Meliaceae/NX0169/W/T	Whole plants	Drying	Oral	Abdominal Pain D01 (100) Asthma R96 (94) Diarrhoea D11 (96)
Lang Du	Lei Bu Ne Du	*Stellera chamaejasme* Linn.	Thymelaeaceae/NX0077/W/H	Roots	Crushing	Oral	Constipation D12 (85)
Lan Cang Rao Hua	Wai De	*Wikstroemia delavayi* Lec.	Thymelaeaceae/NX0066, NX0660/W/S	Whole plants, flowers or bark	Crushing	Oral	Epilepsy N88 (23)
Wu Jing	A Ke	*Brassica rapa* Linn.	Brassicaceae/NX0761/C/H	Roots	Drying	Oral	Bladder Symptom U13 (42)
Tong Qiao She Gu	Mu Gu Xu	*Balanophora involucrata* Hook. f. et Thomson	Balanophoraceae/NX0502, NX0686/W/H	Whole plants	Soaking/drying	Oral	Neoplasm of Eye/Adnexa F74 (23) Orchitis/Epididymitis Y74 (85) Trauma/Injury A80 (36) Viral Hepatitis D72 (27)
San Chun Shui Bai Zhi	Ji Xiu	*Myricaria paniculata* P. Y. Zhang et Y. J. Zhang	Tamaricaceae/NX0197, NX0717/W/S	Branches, leaves	Drying	Oral/Topical	Osteoarthrosis other L91 (103) Rash Localized S06 (100) Rheumatoid/Seropositive Arthritis L88 (98)
Jin Qiao Mai	Ruo A Kao Ken	*Fagopyrum dibotrys* (D. Don) Hara	Polygonaceae/NX0490, NX0528/W/H	Roots	Crushing/drying	Oral	Abdominal Pain Epigastric D02 (69) Hair/Scalp Symptom S24 (65) Heartburn D03 (78) Mumps D71 (90) Peptic Ulcer other D86 (49)
Huo Tan Mu	Zei Lan Xu	*Polygonum chinense* Linn.	Polygonaceae/NX0708/C, W/H	Whole plants	Drying	Oral	Cholecystitis/Cholelithiasis D98 (61)
Dian Bian Da Huang	Lu Zei Ken	*Rheum delavayi* Franch.	Polygonaceae/NX0353/W/H	Roots	Drying/fresh	Oral/Topical	Acute Bronchitis/Bronchiolitis R78 (89) Acute Hepatitis A D73 (103) Bleeding/Haemorrhage NOS A10 (26) Chronic Bronchitis R79 (104) Gastrointestinal Infection D70 (71) Haematuria U06 (59) Heartburn D03 (99) Influenza R80 (104) Pneumonia R81 (104) Upper Respiratory Infection Acute R74 (97)
Li Jiang Da Huang	Ai San Qi	*Rheum likiangense* Sam.	Polygonaceae/NX0262, NX0693/W/H	Roots	Soaking/drying	Oral	Anal Fissure/Perianal Abscess D95 (85) Bleeding/Haemorrhage NOS A10 (95) Bursitis/Tendinitis/Synovitis NOS L87 (73) Gonorrhoea Female X71 (38) Lump/Swelling Localized S04 (85) Melaena D15 (74) Neck Symptom L01 (88) Pain/Tenderness of Skin S01 (78) Pain General/Multiple Sites A01 (83) Rectal Bleeding D16 (100) Throat Symptom R21 (58) Trauma/Injury A80 (79) Viral Hepatitis D72 (79)
Yao Yong Da Huang	Hua Zei De	*Rheum officinale* Baill.	Polygonaceae/NX0753/C, W/H	Roots	Drying	Oral	Constipation D12 (104) Diarrhoea D11 (104) Gonorrhoea Female X71 (104)
Ni Bo Er Suan Mo	Hua Leng Hua Zei Ke	*Rumex nepalensis* Spreng.	Polygonaceae/NX0074/W/H	Roots	Drying/fresh	Oral/Topical	Constipation D12 (84) Pruritus S02 (36) Worms/Other Parasites D96 (90)
Jin Tie Suo	Du La Pei	*Psammosilene tunicoides* W. C. Wu et C. Y. Wu	Caryophyllaceae/NX0488/W/H	Roots	Crushing/soaking	Topical	Abdominal Pain Epigastric D02 (35) Bleeding/Hemorrhage NOS A10 (103) Musculoskeletal Disease other L99 (104) Osteoarthrosis other L91 (101) Pain General/Multiple Sites A01 (104) Rheumatoid/Seropositive Arthritis L88 (104) Trauma/Injury A80 (98)
Qian Sui Gu	Mei Ru	*Amaranthus hypochondriacus* Linn.	Amaranthaceae/NX0525/W/H	Seeds	Drying	Oral	Dyspepsia/Indigestion D07 (39) Sleep Disturbance P06 (24)
Li Guo Xian Ren Zhang	Cong Hei	*Opuntia ficus*-*indica* (Linn.) Mill.	Cactaceae/NX0109/C, W/H	Whole plants	Fresh	Topical	Burn/Scald S14 (99) Gonorrhoea Male Y71 (63)
Jun Qian Zi	Tao Zhi	*Diospyros lotus* Linn.	Ebenaceae/NX0170/C, W/T	Fruits	Drying	Oral	Diarrhoea D11 (86)
Pu Tong Lu Ti Cao	Jiu Gu Lei	*Pyrola decorata* H. Andr.	Ericaceae/NX0152, NX0652/W/H	Whole plants	Drying	Oral	Abdominal Pain Localized other D06 (86) Acute Bronchitis/Bronchiolitis R78 (95) Chronic Bronchitis R79 (79) Influenza R80 (700) Mouth/Tongue/Lip Symptom D20 (79) Upper Respiratory Infection Acute R74 (69)
Ye Hua Du Juan	Shua Dai Lan Ba	*Rhododendron racemosum* Franch.	Ericaceae/NX0085/W/S	Branches, flowers	Crushing	Topical	Psoriasis S91 (34)
Huang Bei Du Juan	Mu Gou Ba Shi	*Rhododendron wardii* W. W. Sm.	Ericaceae/NX0310, NX0312/W/T	Flowers, fruits	Crushing/drying	Oral/Topical	Musculoskeletal Disease other L99 (87) Osteoarthrosis other L91 (76) Rheumatoid/Seropositive Arthritis L88 (82)
Dian Long Dan Cao	Ji Ka	*Gentiana rigescens* Franch. ex Hemsl.	Gentianaceae/NX0350/W/H	Whole plants	Crushing/soak	Oral	Cholecystitis/Cholelithiasis D98 (104) Viral Hepatitis D72 (104)
Xi Nan Cu Kang Shu	Nu Ao	*Ehretia corylifolia* C. H. Wright	Boraginaceae/NX0111/W/T	Whole plants	Soaking	Topical	Pruritus S02 (35)
Ye Ba Zi	Ke Du	*Elsholtzia rugulosa* Hemsl.	Lamiaceae/NX0178/W/H	Leaves, flowers	Crushing/drying	Oral	Influenza R80 (77) Upper Respiratory Infection Acute R74 (104)
Li Jiang Huang Qin	Bai Qi Ba Pei Ke	*Scutellaria likiangensis* Diels	Lamiaceae/NX0696/W/H	Roots	Soak/drying	Oral/Buccal	Swallowing Problem D21 (95)
Bian Da Xiu Qiu	A You Jian Da Ke	*Hemiphragma heterophyllum* Wall.	Plantaginaceae/NX0228/W/H	Whole plants	Drying	Oral	Low Back Symptom L03 (75) Menstruation Irregular/Frequent X07 (33) Musculoskeletal Disease other L99 (69) Osteoarthrosis other L91 (88) Pain General/Multiple Sites A01 (75)
Kuan Ye Tu Er Feng	Du Mei Gu Fu Pie	*Ainsliaea latifolia* (D. Don) Sch.-Bip.	Asteraceae/NX0098/W/H	Whole plants	Drying	Oral	Cough R05 (104) Haemoptysis R24 (82) Malaria A73 (36) Rheumatoid/Seropositive Arthritis L88 (87)
Niu Wei Hao	Qi Ai	*Artemisia dubia* Wall. ex Bess.	Asteraceae/NX0707/W/H	Stem, leaves	Soaking	Topical	Menstruation Absent/Scanty X05 (53)
Nan Ai Hao	Beng Pei	*Artemisia verlotorum* Lam.	Asteraceae/NX0358, NX0658/W/H	Above-ground part	Crushing/soaking/drying	Oral/Topical	Anal Fissure/Perianal Abscess D95 (45) Influenza R80 (104) Upper Respiratory Infection Acute R74 (75)
Yun Nan Hao	Beng Na	*Artemisia yunnanensis* J. F. Jeffrey ex Diels	Asteraceae/NX0618/W/H	Branches, leaves	Crushing	Topical	Nose Bleed/Epistaxis R06 (68)
Wu Jing Huan Yang Shen	Ze Ge	*Crepis napifera* (Franch.) Babc.	Asteraceae/NX0748/W/H	Roots	Fresh	Topical/Oral	Genital Disease Male other Y99 (46) Visual Disturbance other F05 (25) Whooping Cough R71 (101)
Da Ding Cao	Jiu Ban Er	*Gerbera anandria* (Linn.) Sch.-Bip.	Asteraceae/NX0464/W/H	Whole plants	Crushing/drying	Oral	Gonorrhoea Female X71 (23) Worms/Other Parasites D96 (52)
Jie Gu Mu	Su Kua Na	*Sambucus williamsii* Hance	Adoxaceae/NX0049/C, W/S	Bark	Crushing	Topical	Fracture: Femur L75 (95) Fracture: Hand/Foot Bone L74 (86)Fracture: Other L76 (78) Fracture: Radius/Ulna L72 (77) Fracture: Tibia/Fibula L73 (89) Low Back Symptom L03 (63) Osteoarthrosis other L91 (58) Pain General/Multiple Sites A01 (58) Rheumatoid/Seropositive Arthritis L88 (101)
Zhu Zi Shen	Man Hai Lü	*Panax japonicus* var. *major* (Burkill) C. Y. Wu et K. M. Feng	Araliaceae/NX0536, NX0736/C,W/H	Roots	Crushing	Oral	Elevated Blood Pressure K85 (96) Genital Disease Male other Y99 (29) Low Back Symptom L03 (84) Pain General/Multiple Sites A01 (104) Trauma/Injury A80 (104)
Chuan Dian Chai Hu	Mu Ru	*Bupleurum candollei* Wall. ex DC.	Apiaceae/NX0453/W/H	Whole plants	Drying	Oral	Influenza R80 (100) Pneumonia R81 (86) Upper Respiratory Infection Acute R74 (104)
Hui Xiang	Lai Wu Ci E	*Foeniculum vulgare* Mill.	Apiaceae/NX0108/C/H	Whole plants	Fresh	Oral	Abdominal Pain Localized other D06 (46) Bedwetting/Enuresis P12 (24) Cystitis/Urinary Infection other U71 (77)Gonorrhoea Female X71 (23) Orchitis/Epididymitis Y74 (31) Urinary Frequency/Urgency U02 (101)
Bai Liang Du Huo	Guo Ru Ke	*Heracleum candicans* Wall. ex DC.	Apiaceae/NX0334/W/H	Roots	Crushing/drying	Oral	Abdominal Pain D01 (86) Abdominal Pain Epigastric D02 (69) Cough R05 (58)

Angiosperms are sorted follows APG IV

^a^Habitat: W, wild; C, cultivated

^b^Habit: H: herbs; T: trees; S: shrubs; WV: woody vines; CS: climbing shrubs; HV: herbal vines; M: Macrofungi

Further analysis indicated that most of the plant species were utilized for the group of digestive diseases (D; Nur=36, Nt=15), followed by the respiratory (R; Nur=29, Nt=13,), musculoskeletal (L; Nur=21, Nt=12), and the general and unspecified disease group (A; Nur=21, Nt=5). The ICF values of these four disease groups were low: Group D: 0.60, Group R: 0.57, group L: 0.45, and group A: 0.75. These low values indicated that these four groups of diseases are common diseases in Naxi people living areas, and Naxi folk healers have a high consensus on the treatment of these diseases.

For the treatment of diabetes (T89: Diabetes Insulin Dependent or T90: Diabetes Non-Insulin Dependent), which is an endocrine disease that belongs to the group of endocrine/metabolic and nutritional, only one plant species was cited in the Dongba Sutras. *Diaphragma juglandis fructus*, the dry wood diaphragm tissue (xylem septa) that grows inside the walnut (*Juglans regia*), was reported as a medicinal plant that can be used to treat diabetes, and the consensus on this treatment was high. A previous study reported that the flavonoids from *Diaphragma juglandis fructus* have significant anti-diabetic activity [29]. This shows that as the knowledge on folk medicine is collected from long-term practical experience, its scientific nature has yet to be proven by modern science. With more research, more information from traditional medicinal practices will be scientifically proven.

The plant species with the highest number of use reports were *Rheum likiangense* (13 use reports), *Reineckea carnea* (11 use reports), *Rheum delavayi* (10 use reports), and *Hypericum augustinii* (10 use reports). *Rheum likiangense* and *R. delavayi* are endemic to a small district, and *Reineckea carnea and Hypericum augustinii* are endemic to China. This emphasizes the uniqueness of Naxi medicinal plants.

### Analysis of endemic species

**Fig. 2:**
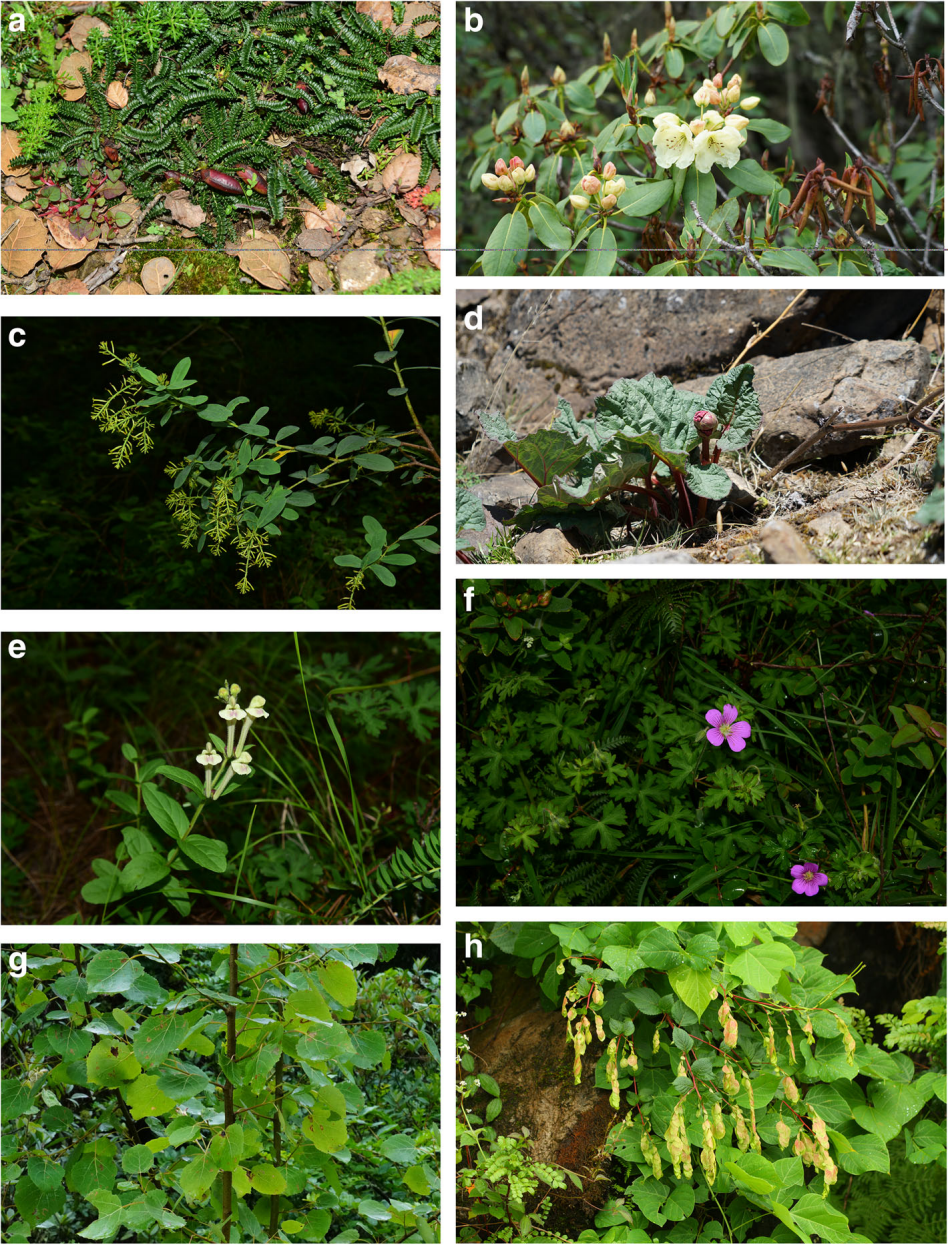
Eight plant species endemic to China present in the areas inhabited by Naxi people. **a**: *Chesneya polystichoides*; **b**: *Rhododendron wardii*; **c**: *Wikstroemia delavayi*; **d**: *Rheum likiangense*; **e**: *Scutellaria likiangensis*; **f**: *Geranium strictipes*; **g**: *Populus rotundifolia* var. *bonatii*; **h**: *Dobinea delavayi*

Among the medicinal plants in the Dongba Sutras, 25 species are endemic to China, accounting for 29.41% of the total number of medicinal plant species in the Dongba Sutras (85 species) (Table 10). Moreover, there are eight species only distributed in the areas inhabited by Naxi people (Fig. 2), including northwest Yunnan, southwest Sichuan, and Southeast Tibet. Examples include *Populus rotundifolia* var. *bonatii*, *Rheum likiangense*, *Chesneya polystichoides*, *Geranium strictipes*, *Dobinea delavayi*, *Wikstroemia delavayi*, *Rhododendron wardii*, and *Scutellaria likiangensis.*

**Table 10:** Chinese endemic plant species recorded in the Dongba Sutras

ID	Family	Scientific name	Distribution*	Abundance**	Resource type
1	Anacardiaceae	*Dobinea delavayi*	**SW**	Sol	Wild
2	Boraginaceae	*Ehretia corylifolia*	SW	Cop1	Wild
3	Caryophyllaceae	*Psammosilene tunicoides*	SW	Sp	Wild
4	Compositae	*Artemisia yunnanensis*	SW,W	Cop2	Wild
5	Compositae	*Crepis napifera*	SW	Cop2	Wild
6	Cupressaceae	*Cupressus duclouxiana*	SW	Cop1	Wild
7	Ericaceae	*Rhododendron racemosum*	SW	Cop3	Wild
8	Ericaceae	*Rhododendron wardii*	**SW**	Cop1	Wild
9	Geraniaceae	*Geranium strictipes*	**SW**	Cop1	Wild
10	Gramineae	*Fargesia orbiculata*	SW	Cop1	Wild
11	Guttiferae	*Hypericum augustinii*	SW	Cop3	Wild
12	Labiatae	*Scutellaria likiangensis*	**SW**	Cop1	Wild
13	Leguminosae	*Chesneya polystichoides*	**SW**	Sp	Wild
14	Liliaceae	*Asparagus meioclados*	SW	Cop1	Wild
15	Magnoliaceae	*Magnolia delavayi*	SW	Sp	Cultivated
16	Pinaceae	*Pinus yunnanensis*	SW,S	Soe	Wild
17	Polygonaceae	*Rheum likiangense*	**SW**	Sp	Wild
18	Polygonaceae	*Rheum officinale*	SW,S,C	Cop2	Cultivated,wild
19	Rosaceae	*Rubus coreanus* var. *tomentosus*	SW,C,W	Cop2	Wild
20	Sabiaceae	*Meliosma cuneifolia*	SW,C,W	Cop1	Wild
21	Salicaceae	*Populus rotundifolia* var. *bonatii*	**SW**	Soe	Wild
22	Salicaceae	*Salix variegata*	SW,C,W	Cop3	Wild
23	Sapindaceae	*Sapindus delavayi*	SW,C	Sp	Cultivated,wild
24	Tamaricaceae	*Myricaria paniculata*	SW,C,W	Cop1	Wild
25	Thymelaeaceae	*Wikstroemia delavayi*	**SW**	Cop1	Wild

*Note:SW–Southwest China; C–Central China; W–West China; S–South China

**Soe (Sociales): High number of individuals, the above-ground plant part is closed

Cop3 (Copiosae): High number of individuals, but the above-ground plant part is not closed

Cop2: Large and common plants

Cop1: Large plants, but small populations

Sp (Sparsal): Low number of plants, scattered

Sol (Solitariae): Low number of plants, sparse

Un (Unicum): Only one individual

The Naxi people consider human beings and nature as brothers. This ecological ethics concept lays the foundation for the Naxi people to live in harmony with nature; it shows the most primitive and simple concept of environmental conservation by human beings [30]. The distribution area of these plant species is very small. Although the Naxi people have been using these plants as medicinal materials for a long time, their populations are still stable, indicating that Naxi people attach great importance to plant conservation when collecting these medicinal plants. The Naxi people collect medicinal materials from their surroundings to treat many diseases. They never harm the environment during plant collecting, and they are grateful for being able to take advantage of wild medicinal plants. This fully embodies their idea of maintaining ecological balance. Meanwhile, artificial cultivation was adapted to expand the population of medicinal plants with rare natural resources in order to minimize their impact on wild plant resources.

## Conclusions

### A variety of herbal medicine was recorded in the Dongba sutras

The medicinal plants used by the Naxi people are diverse. A variety of herbal medicine closely related to the life of the Naxi people was recorded in the Dongba Sutras. A total of 85 species of medicinal plants and fungi belonging to 51 families and 71 genera were recorded in the Dongba Sutras, among which 25 species are endemic to China, and 8 species are distributed in a small region. There were 22 species of medicinal dietary plants recorded in the Dongba Sutras.

### The basic features of traditional Naxi medicine

The knowledge of traditional Naxi medicine is always in the hands of the elderly and clergy. The traditional apprenticeship between the elderly and the young makes an assurance of the knowledge inheritance from age to age. Dongba, as the clergyman in the Naxi people, records the most important medical knowledge in the Dongba Sutras for better inheritance.

In the processing of medicinal materials, Naxi people make good use of fresh products, medicinal liquids, and plant powders. No complex processing is required from the raw plants to the medicine used, which is very convenient. Medicinal liquids can fully dissolve alcohol-soluble active substances and are easy to store. Different types of mixed powder are used internally or externally suiting the remedy to the different cases, which not only brings convenience to clinical uses but also protects the intellectual property rights of the folk healers because it is hard to know which medicinal plants are used in the powders.

The Naxi ancestors inhabit mountainous areas and are seldom influenced by alien cultures. As a result, the methods of medication are easy to follow, mainly including decocting, oral consumption with warm water, and topical. And the processing technology of Naxi medicine only includes some simple procedures like washing, drying, and crushing.

Four groups of diseases are common diseases in Naxi people living areas: they are the group of digestive diseases (D), followed by the respiratory (R), musculoskeletal (L), and the general and unspecified disease group (A). The Naxi folk healers have a high consensus on the treatment of these diseases.

### The ecological ethics of Naxi people have positive significance for the conservation of wild plant resources

Hengduan mountainous where the Naxi people who live own one of the greatest abundant biodiversities in the world. Naxi people always keep the scientific ecological ethics concept in mind. The Naxi people never harm the environment during plant collecting, and they are grateful for being able to take advantage of wild medicinal plants. Meanwhile, artificial cultivation is adapted to expand the population of medicinal plants with rare natural resources in order to minimize their impact on wild plant resources.

**Fig 3:**
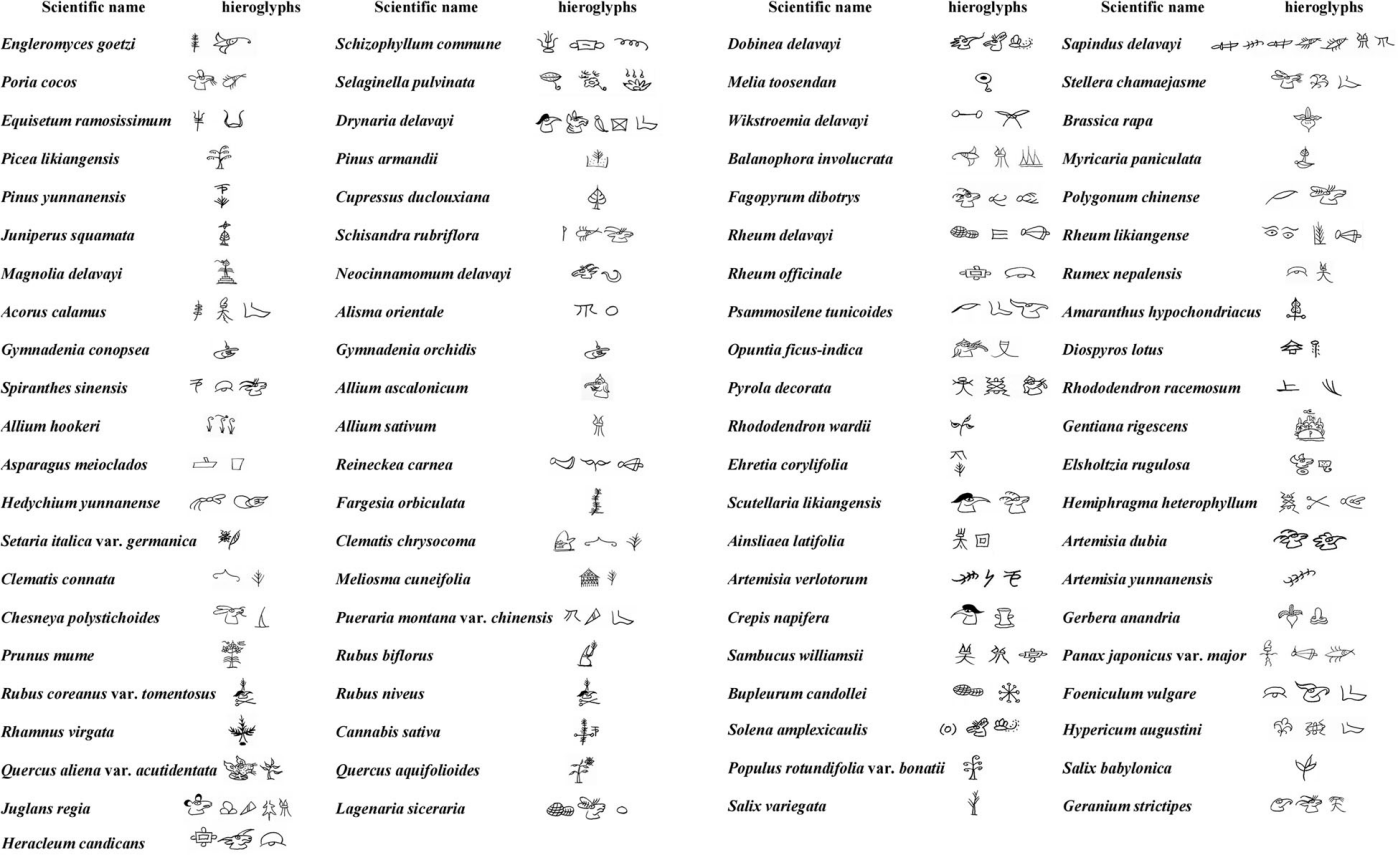
85 Medicinal plants and fungi with hieroglyphs in Dongba Sutras

Dongba Sutras are recorded in hieroglyphics (Fig. 3); thus, only the Dongbas, as the clergymen, can fully understand them. Contents of the Dongba Sutras are all-encompassing. Medical knowledge only takes a small part of the whole contents, and the records are not comprehensive enough. In addition, the folk medicinal knowledge is orally passed down. Thus, it is necessary to further deepen the investigation and research efforts to systematically organize and catalog the Naxi people’s unique traditional medicine, exhibiting its due brilliance.

## Data Availability

All data generated or analyzed during this study are included in this published article (and its supplementary information files).

## Abbreviations

APG IV: The Angiosperm Phylogeny Group classification for the orders and families of flowering plants ed.IV
AQSIQ: General Administration of Quality Supervision of China
IMDY: The Herbarium, Yunnan Branch, Institute of Medicinal Plants, Chinese Academy of Medical Science
ICF: The informant consensus factor
ICPC-2: International Classification of Primary Care, revised second ed
WONCA: World Organization of Family Doctors
WHO: World Health Organization
